# A pre and post evaluation of the communication and interaction training programme for professionals in dementia care

**DOI:** 10.1111/papt.12568

**Published:** 2024-12-19

**Authors:** Sophie Trees, Ian Andrew James, Sally Stapleton, Daniel Rippon

**Affiliations:** ^1^ Sussex Partnership NHS Foundation Trust Sussex UK; ^2^ Cumbria, Northumberland, Tyne and Wear NHS Foundation Trust Newcastle Upon Tyne UK; ^3^ Northumbria University Newcastle Upon Tyne UK

**Keywords:** communication, dementia, healthcare professional, interaction

## Abstract

**Background:**

NICE guidelines advocate that healthcare professionals should aim to use non‐pharmacological and person‐centred approaches as primary strategies to reduce or prevent distress in people living with dementia who reside within care settings. However, despite these recommendations, recent studies have illustrated that there is still a requirement for healthcare professionals to have adequate opportunities to access training programmes and guidance on how to effectively use non‐pharmacological approaches in dementia care settings. Communication and Interaction Training (CAIT) was developed to train healthcare professionals in dementia care on how to apply person‐centred principles to effectively reduce or negate distress in people living with dementia in a non‐invasive manner.

**Aims:**

This paper provides an overview of current debates regarding the use of non‐pharmacological approaches in dementia care, as initial care strategies, to reduce the primary use of pharmacological interventions that may have deleterious side effects for people living with dementia. Furthermore, this paper provides a summary of an evaluation that assessed the extent to which a 2‐day CAIT programme could enhance healthcare professionals in their perceived ability to communicate therapeutically with and provide care for people living with dementia.

**Materials & Methods:**

In this evaluation, 35 healthcare professionals in dementia care engaged with the 2‐day CAIT programme. The Confidence in Dementia Scale, Knowledge in Dementia Scale and Compassionate Competence Scale were administered for participants to complete pre and post training.

**Results:**

A series of parametric paired samples *t*‐tests were completed, and the results indicated that the 2‐day CAIT course was effective in enhancing healthcare professionals' perceived confidence, communication skills, sensitivity, and ability to meet the care needs of people living with dementia. However, staff’ knowledge of dementia did not significantly increase following the CAIT course, which could be due to participants already having high levels of knowledge on dementia prior to training.

**Discussion & Conclusion:**

These results indicated that engaging in CAIT could be beneficial in enhancing healthcare professionals' perceived ability to use therapeutic communication strategies in their interactions with people living with dementia. Discussion is provided on how the delivery of training programmes, such as CAIT, may assist in re‐enforcing guidelines that advocate for the use of non‐pharmacological and non‐invasive approaches in dementia care.

## INTRODUCTION

People living with dementia can experience various symptoms, such as disorientation in time and place, agitation, repetitive vocalisations, and sleep disturbances (Mukherjee et al., 2017). It has been recognised that people living with dementia can have difficulties in verbally communicating their needs, which can potentially reduce quality of life and the capacity to complete activities of daily living (ADLs) independently (Kales et al., 2014). Reductions in the capacity to complete ADLs, such as food preparation, dressing and bathing, can potentially require people living with dementia to receive care within hospital inpatient ward (Veedfald et al., 2021) or longer term care home settings (Johansen et al., 2020). The process of being admitted into dementia care settings, such as hospital wards and care homes, can be challenging and potentially have negative consequences for people living with dementia. For example, the unfamiliarity of acute ward settings can exacerbate disorientation, increase risk of falls and further impair cognitive functioning for people living with dementia (Dewing & Dijk, 2016). Admission into long‐term care home settings can also be a risk factor for loneliness (Hanratty et al., 2018), sleep disturbances (Webster et al., 2020) and reduced mood (Aggarwal et al., 2022). According to Kitwood (1997), it is necessary for dementia care settings to facilitate people living with dementia to achieve and maintain the following person‐centred care needs; (1) comfort, (2) engagement in activities that align with personal interests, (3) social inclusion, (4) opportunities to interact with others and (5) maintenance of personal identity. Healthcare professionals, such as nurses, healthcare assistants and doctors, have been recognised as being pivotal in the successful delivery of person‐centred care in dementia care settings (Blake et al., 2020). However, healthcare professionals can experience various challenges in the delivery of person‐centred care and gaining an understanding of any unmet needs that people living with dementia may encounter. For example, communication strategies that includes asking a care recipient to verbally communicate their healthcare needs may be suboptimal in identifying any unmet needs when interacting with people living with dementia who have cognitive impairments (Allwood et al., 2017). People living with dementia who experience impairments to speech/language and disorientation in time/place can encounter difficulties in communicating their person centred needs to healthcare professionals (Banovic et al., 2018). Furthermore, when people living with dementia are unable to communicate and achieve particular aspects of these person‐centred needs when residing in dementia care settings, this can then manifest into overt distress (Cohen‐Mansfield et al., 2015). Distress can manifest as overt agitation, repetitive vocalisations and social withdrawal (Cohen‐Mansfield, 2000). Thus, it is necessary to acknowledge that overt signs of distress can be a method for people living with dementia to communicate unmet needs (Pritchard & Dening, 2022).

Historically, pharmacological interventions such as atypical antipsychotics, benzodiazepines, donepezil and galantamine, have been prescribed to reduce distress and agitation in people who have dementia (Dyer et al., 2018). However, there have been calls to reduce or negate the use of pharmacological interventions for people living with dementia who exhibit distress due to their adverse side effects, such as drowsiness, hypotension, and cardiotoxicity (Banerjee, 2009; Gray et al., 2021). Furthermore, it has been argued that the use of pharmacological interventions may not be effective in addressing and meeting the person‐centred care needs of people living with dementia (Magierski et al., 2020). However, it has been posited that lack of opportunities to engage in relevant training programmes may perpetuate the use of pharmacological/physical restraint and thwart the application of more therapeutic approaches in meeting the person‐centred care needs of people who reside in dementia care settings (Wong et al., 2024).

Therefore, it has been advocated that dementia care services support healthcare professionals to employ non‐pharmacological approaches in their routine care practices as primary strategies to meet the person‐centred care needs of people living with dementia in order to ameliorate or prevent the onset of distress (Ballard et al., 2020). However, there is a limited evidence base to support and guide healthcare professionals on the safe application of non‐pharmacological approaches in dementia care settings (Gray et al., 2021; James & Moniz‐Cook, 2022). It has been argued that there is a necessity for the further development and delivery of training programmes that aim to guide healthcare professionals on the use of non‐pharmacological approaches to meet the psychosocial needs of people living with dementia (O'Donnell et al., 2022). Training programmes that focus on enhancing the communication skills of healthcare professionals when providing care for people living with dementia has been illustrated as an effective way to facilitate the use of non‐pharmacological and non‐invasive approaches in ameliorating distress (Livingston et al., 2014). A systematic review has also indicated that engaging in training programmes that aim to enhance healthcare professionals' communication skills can be beneficial in facilitating their knowledge and understanding of identifying any unmet needs that may cause distress for people living with dementia (Nguyen et al., 2019). Furthermore, the process of enhancing healthcare professionals' confidence in using non‐pharmacological approaches in identifying and meeting the care needs of people living with dementia can also enhance the delivery of care and negate the onset of distress in care recipients (Crandall et al., 2022).

Thus, the development of the Communication and Interaction Training programme (CAIT) has aimed to support and educate healthcare professionals on how to apply non‐pharmacological practices and communicate therapeutically with people living with dementia (James & Gibbons, 2019). CAIT is recommended as an approach within the Division of Clinical Psychology Guidelines on the use of non‐pharmacological approaches and therapeutic interactions in dementia care settings (James et al., 2024). CAIT is constructed on the principles of person‐centred care, and its development has been informed by previous training programmes on effective ways of communicating in healthcare settings (Dorey et al., 2019; Eggenberger et al., 2013; Harwood et al., 2018). The CAIT programme is a compendium of evidence‐based tools aimed at facilitating therapeutic communication and interactions between healthcare professionals and people living with dementia (James et al., 2022). The CAIT programme also acknowledges healthcare professionals as ‘experts by experience’ and has been designed to facilitate reflective practice. Hence, the programme builds on healthcare professionals' existing skills and experiences, endeavouring to better structure and fine‐tune their competencies in supporting people living with dementia; with such an approach being found to have an empowering effect and enhancement of carer self‐efficacy (James & Gibbons, 2019). This latter aspect of CAIT is particularly pertinent as occupational support that facilitates higher levels of perceived competence and self‐efficacy has been associated with lower caregiver strain in healthcare professionals who provide care for people living with dementia (Karantzas et al., 2016). Furthermore, it has been acknowledged that higher levels of self‐efficacy can facilitate healthcare professionals in successfully supporting people living with dementia using non‐pharmacological methods (Hughes et al., 2008). Thus, it is of interest to assess the extent to which the CAIT programme can enhance healthcare professionals in their perceived confidence and ability to communicate with people diagnosed with a dementia.

It has been acknowledged that the provision of support and training that aims to enhance healthcare professionals' ability to identify unmet needs of people living with dementia may facilitate the use of non‐pharmacological approaches and ensure therapeutic interactions between healthcare professionals and care recipients in dementia care settings (Nguyen et al., 2022). The CAIT programme has been trialled and piloted with healthcare professionals in dementia care where participant feedback has informed the further refinement of training packages to support frontline staff in their continual professional development and enhancement of communication skills (James & Gibbons, 2019; James & Stapleton, 2023). CAIT has now been rolled out across the UK and on an international level for healthcare professionals who provide care in various dementia care settings, including inpatient wards and care homes, in order to enable frontline staff to access the training programme (Reichelt et al., 2023). This study aimed to evaluate the effectiveness of a 2‐day CAIT programme on enhancing healthcare professionals' knowledge, confidence and perceived communication skills when providing direct care for people living with dementia. This study was part of a wider project that aimed to support healthcare professionals on the safe and effective application of non‐pharmacological interventions to reduce or negate the use of restrictive interventions in dementia care settings.

The following hypotheses were tested:

1. Engaging in the 2‐day CAIT programme would significantly increase participants' sense of confidence to deliver and apply non‐pharmacological approaches in dementia care settings.
2. Engagement in the 2‐day CAIT programme would significantly increase participants perceived efficacy to communicate therapeutically with people living with dementia.
3. Engagement in the 2‐day CAIT programme would significantly increase perceived sensitivity in identifying and meeting the healthcare needs of people living with dementia.
4. The 2‐day CAIT programme would enhance participants' perceived insights and ability to deliver person‐centred care for people who have dementia.
5. The 2‐day CAIT programme would improve participants' knowledge of dementia.

## METHOD

### Design

A pre‐post intervention design was employed in this study. This type of research design has been used in previous studies that have evaluated training programmes for healthcare professionals working in dementia care settings (Elvish et al., 2018; Reichelt et al., 2023). A series of paired samples *t*‐tests were conducted to assess if improvements were reported in participants' perceived confidence in care delivery, knowledge of dementia, efficacy in communication, sensitivity to and identification of care recipients' needs following the 2‐day CAIT programme.

### Participants

**FIGURE 1:**
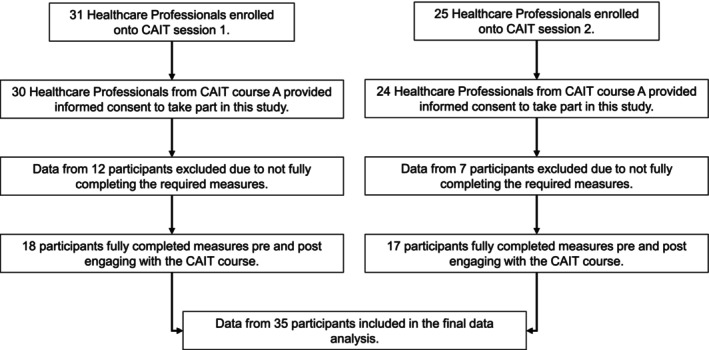
Flowchart illustrating the attrition of data and number of participants who fully completed the measures as required for the present study.

Participants were recruited via an integrated care system for dementia care that included care partners within the statutory and charitable sector. Details of the 2‐day CAIT course were distributed to locality managers of dementia care services within an integrated care system to promote healthcare professionals to engage with the training programme. There were 2 separate CAIT sessions that were offered for dementia healthcare professionals to engage with. There were 31 healthcare professionals who enrolled onto the first CAIT session and 25 healthcare professionals who engaged with the second CAIT session. Both of the courses were delivered by the same facilitator. Participants either had previously expressed an interest to engage with CAIT or had been selected by locality managers as being healthcare professionals in key positions who could facilitate the role out of CAIT in their locality. Participants who enrolled onto the 2‐day CAIT course were then invited to take part in this study as part of an evaluation of the training delivery. As part of this invite, participants were provided with an information sheet that detailed the aims and what participation in the study would require to ensure that informed consent could be obtained prior to any data collection. There were 54 out of the 56 healthcare professionals who agreed to take part in the present study. Participants who provided informed consent to take part in this study were healthcare professionals within dementia care settings, including Clinical Psychologists, Nurses, and Occupational Therapists. However, data from 19 of the participants were excluded from the statistical analysis due to measures not being fully completed. Therefore, in total, there were 35 healthcare professionals in dementia care who engaged in the 2‐day CAIT course and fully participated in the present study. Details of participant attrition of data are illustrated in Figure 1.

### Measures

The current evaluation used an online survey platform consisting of demographic data (age, gender identity, job title and years of experience in professional dementia care) and three measures; Confidence in Dementia Scale (CODE), Knowledge in Dementia Scale (KIDE) (Elvish et al., 2014) and Compassionate Competence Scale (CCS) (Lee & Seomun, 2016). Participants were asked to complete the stipulated measures and provide details of demographic information (age, gender identity and job title) at the start of day 1 of the 2‐day CAIT course and before any content had been delivered. Participants were also required to complete the CODE, KIDE and CCS at the end of day 2 of the CAIT course after all of the content had been delivered.

### Confidence in dementia scale

The CODE (CODE, Elvish et al., 2014) includes 9 items and measures perceived confidence in providing care for people living with dementia. This measure includes items such as “I feel able to understand the needs of a person with dementia when they cannot communicate well verbally” and “I feel able to interact with a person with dementia when they cannot communicate well verbally”. Participants were required to respond to each item on a 5‐point Likert scale from ‘not able’ to ‘very able’, with overall scores ranging from 9 to 45. Higher scores indicate greater levels of participants' perceived confidence in their provision of healthcare for people living with dementia. This measure has been found to have good internal consistency (*α* = .89; .91) (Elvish et al., 2014, 2018). Similar evaluations of dementia‐specific training for staff have also used the CODE (Reichelt et al., 2023).

### Knowledge in dementia scale

The KIDE (KIDE, Elvish et al., 2014) comprises of 16 items, such as “People living with dementia will eventually lose all their ability to communicate” and “My perception of reality may be different from that of a person with dementia”. Participants were required to respond either agree or disagree to each of the items. Scores can range from 0 to 16, with a higher score indicating greater knowledge of dementia. This measure has been found to have good internal consistency (*α* = .66–.72) (Elvish et al., 2014, 2018). Other evaluations of dementia‐specific training for healthcare staff professionals used the KIDE to assess dementia related knowledge of participants (Elvish et al., 2018).

### Compassion competence scale

The Compassion Competence Scale (Lee & Seomun, 2016) is a 17‐item self‐report questionnaire with three subscales: communication, sensitivity and insight. The communication subscale consists of 8 items and assesses the perceived ability to communicate with care recipients in a therapeutic manner. The sensitivity subscale consists of 5 items that assess the perceived ability to identify and respond to emotional changes in care recipients. The insight subscale includes four items and assesses comprehensive awareness and perceived ability to understand and identify the unmet needs of care recipients who may have difficulties in verbally communicating their needs. Responses are measured on a 5‐point Likert scale from ‘strongly agree’ to ‘strongly disagree’, with a higher score on each subscale indicating higher levels of perceived communication skills, sensitivity, and insight towards care recipients. The measure was developed for use with nursing staff more broadly, however, it has since been used in a pre‐post study design specifically for healthcare professionals in dementia care (Bennett et al., 2022). This measure was found to have good internal consistency both as an overall measure (*α* = .91) and for the three subscales: Communication (*α* = .88), Sensitivity (*α* = .77) and Insight (*α* = .73) (Lee & Seomun, 2016).

### Ethical considerations

This study obtained ethical approval from the ethics committee at the School of Health and Life Sciences, Northumbria University (REF 49618).

### Procedure and details of training

Both of the 2‐day CAIT courses were delivered in a dedicated educational suite of a learning and development centre at a hospital site. Each of the CAIT courses were delivered over the course of 2 consecutive days. The training was delivered by the developer of the course on a face‐to‐face basis where participants were physically present at the location of training delivery. Participants were asked to scan a QR code that provided access to the stipulated baseline measures on a Qualtrics platform for completion. The CAIT programme is composed of six sections: (1) Descriptions of Dementia, including cognitive and sensory changes that can impact the communication; (2) Description of stress, distress and agitation, and reframing them as potential forms of communication; (3) Generic communication skills; (4) Core dementia care related communication skills; (5) Communication skills associated with intimate care tasks, and working in people's intimate spaces; and (6) Use of care plans, formulation, and delivery of complex care. Participants were sat in cabaret style seating plan with tables of six seats throughout the delivery of the CAIT course. Following the delivery of each component of the CAIT course, participants engaged in group discussions, comprising of 5–6 people, to then feedback the content of their discussion to the course facilitator. Table 1 provides an overview of the structure and content of the CAIT programme over the 2 days.

**TABLE 1:** An overview of the 2‐day CAIT programme.

Day 1	Day 2
Overview of CAIT Overview of the CAIT teaching	Nature of Signs of unmet need What are the “Signs of unmet need”?
Functional and sensory changes in dementia Memory Sensory changes Time machine Fast and slow thinking in dementia	Formulations Overview of formulations used in UK Formulation Inpatient formulations
Communication skills for use in dementia care Communication skills 1 Communication skills 2: Pauses and stressing of words Use of therapeutic lies	Prevention and de‐escalation of distress Non‐medication interventions in dementia Namaste Use of Verbal Judo in dementia care
Unmet needs perspective The unmet needs perspective	Last resort strategies Restraint Prevention and Management of Violence & Aggression

At the end of the second day of the 2‐day course (post‐CAIT), participants were asked to complete the CODE, KIDE and CCS measures via a Qualtrics link to enable the analysis of pre and post training data.

### Statistical analysis

Parametric paired samples *t*‐tests were used. *Z*‐scores were calculated to identify outliers, two participants' data contained outliers (*z* > 3) (Field, 2013) and were therefore removed before further analyses. Paired samples *t*‐tests were conducted to compare means between pre‐CAIT and post‐CAIT for (1) CODE, (2) KIDE, and the following subscales of CCS: (3) communication, (4) sensitivity, (5) insight. Effect sizes were calculated using Cohen's d to determine if an effect is small (0.3), medium (0.5) or large (0.8) (Cohen, 1998). The lead author used SPSS to report descriptive statistics and conduct the data analysis. Analysis of data was also conducted by the last‐named author for verification purposes.

## RESULTS

Of the 35 participants, 6 participants identified as male (mean age = 42.33, *SD* = 13.68) and 29 identified as female (mean age = 47.93, *SD* = 8.27). The years of experience in providing healthcare for people living with dementia ranged from 0.5 years to 38 years (*M* = 15.74, *SD* = 10.34). Please refer to Table 2 for the details of the disciplines that the participants worked within dementia care settings.

**TABLE 2:** Details of discipline and job title of participants.

Discipline	Number of participants
Psychology and Psychological Therapies (PPT)	8
Nursing	13
Occupational Therapy	6
Leadership Roles	8

Histograms of pre‐post change scores for CODE, KIDE and the three subscales of CCS indicated the assumption of normality had been met, and therefore parametric paired samples *t*‐tests were used. Paired samples *t*‐tests were conducted to compare means between pre‐CAIT and post‐CAIT for (1) perceived confidence in providing care for people living with dementia, (2) knowledge of dementia and the following subscales of CCS: (3) communication, (4) sensitivity, (5) insight towards the care needs of people living with dementia. Effect sizes were calculated using Cohen's d to determine if an effect size was small (0.3), medium (0.5) or large (0.8) (Cohen, 1988). Table 3 provides details of statistics regarding the pre and post CAIT scores.

**TABLE 3:** A summary of pre and post CAIT scores.

Measure	*n*	Mean (SD)			95% confidence interval of the difference		Significance	Effect size
		Pre	Post	*T*‐value	Lower	Upper	*p* value	Cohen's *d*
CODE	35	35.74 (6.23)	38.74 (4.02)	−3.00	5.03	0.97	.005	0.64
KIDE	35	15.23 (.97)	15.43 (.74)	−1.36	0.50	0.10	.18	0.13
CCS: Communication subscale	35	4.21 (.36)	4.46 (.41)	−4.76	0.36	0.15	<.001	0.75
CCS: Sensitivity subscale	35	4.26 (.48)	4.45 (.55)	−2.47	0.34	0.03	.02	0.45
CCS: Insight subscale	35	4.04 (.59)	4.36 (.54)	−4.70	0.46	0.18	<.001	0.79

A significant increase was observed in participants' perceived confidence to provide effective healthcare for people living with dementia (CODE) between pre (*M* = 35.76, *SD* = 6.23) and post (*M* = 38.74, *SD* = 4.02) CAIT, *t*(34) = −3.00, *p* = .005, and this represented a medium‐sized effect, *d* = 0.64.

On the CCS, participants showed a significant increase between pre (*M* = 4.21, *SD* = 0.36) and post (*M* = 4.46, *SD* = 0.41) CAIT on the communication subscale, *t*(34) = −4.76, *p* = <.001, representing a large‐sized effect, *d* = 0.75. A significant increase between pre (*M* = 4.26, *SD* = 0.48) and post (*M* = 4.45, *SD* = 0.55) CAIT on the sensitivity subscale was observed *t*(34) = −2.47, *p* = .02, representing a medium‐sized effect, *d* = 0.45. A significant increase between pre (*M* = 4.04, *SD* = 0.59) and post (*M* = 4.36, *SD* = 0.54) CAIT was also observed on the insight subscale *t*(34) = −4.70, *p* < .001, representing a large‐sized effect *d* = 0.79. However, there was no significant difference observed in participants' knowledge of dementia between pre (*M* = 15.23, *SD* = 0.97) and post (*M* = 15.43, *SD* = 0.74) CAIT, *t*(34) = −1.36, *p* = .18.

## DISCUSSION

The results indicated that completion of the 2‐day CAIT programme could be beneficial for healthcare professionals in enhancing their perceived confidence to successfully deliver care for people living with dementia. Furthermore, the CAIT programme was also shown to enhance participants' perceived ability to communicate with, be sensitive towards and have awareness of the person‐centred needs of people living with dementia. However, no significant increase in knowledge of dementia was observed following the CAIT programme. It should be noted that the healthcare professionals who took part in the present study were experienced clinicians (an average of 15.74 years) with a high baseline score on the KODE scale. This would suggest that participants had a high level of baseline knowledge on dementia prior to engaging in the CAIT programme.

The findings that CAIT may be beneficial in improving a sense of confidence and self‐efficacy in the delivery of care for people living with dementia could have important implications regarding the occupational well‐being and practice of healthcare professionals. It has been argued that lack of opportunities to engage in training that focuses on facilitating therapeutic communication strategies can potentially prevent healthcare professionals from obtaining opportunities to further enhance confidence in their ability to successfully deliver care for people living with dementia (Zhao et al., 2022). This is concerning given that healthcare professionals who have a low sense of self‐efficacy and confidence in their ability to apply their skills when providing care for people living with dementia can also be vulnerable to the onset of caregiver strain (Karantzas et al., 2016). Furthermore, high levels of caregiver strain can cause healthcare professionals to leave caring professions, which can lead to loss of experience and expertise in some dementia care home settings (Costello et al., 2020). However, a systematic review has indicated that the process of engaging in training programmes that are effective in enhancing healthcare professionals' confidence in communicating therapeutically with people living with dementia can help to reduce or negate caregiver stress and burnout (Conway & Chenery, 2016). It has also been observed that training programmes that facilitate healthcare professionals to use communication strategies that align with the person‐centred care needs of people living dementia can improve attitudes and satisfaction towards the delivery of care practices in dementia care settings (Surr et al., 2016). Thus, it is of interest to conduct further assessments to ascertain if any observed enhancements in perceived confidence in the delivery of dementia care could elicit positive outcomes in the occupational wellbeing of healthcare professionals in dementia care following engagement with the CAIT programme.

The results of the present study also indicated that the 2‐day CAIT programme could enhance healthcare professionals in their perceived ability to communicate therapeutically with people living with dementia. It has been acknowledged that healthcare professionals can encounter difficult and incongruent communication with people living with dementia who are disoriented in time and place (Cohen‐Mansfield, 2000). Thus, there is a necessity for healthcare professionals to have insight and adapt their communication style accordingly to ensure cohesive interactions with people who have dementia, such as approaching care recipients from the front, speaking slowly/clearly and using short/simple sentences during caregiving interactions (Jootun & McGhee, 2011). It has been suggested that training programmes that aim to facilitate and guide healthcare professionals on adapting communication styles in accordance with the bespoke needs of people living with dementia may encourage therapeutic interactions and avoid stressful situations in dementia care settings (Banovic et al., 2018). Furthermore, the process of supporting staff on how to apply therapeutic communication techniques could potentially facilitate healthcare professionals in identifying and meeting the care needs of people living with dementia (Coates & Fossey, 2019). Thus, there is a need for subsequent studies to assess the extent to which any enhanced communication skills and awareness acquired on the CAIT programme transfers into the applied practice of healthcare professionals in dementia care. Furthermore, it would be of interest to ascertain how any enhancements in communications skills, as a result of engaging with the CAIT programme, could facilitate therapeutic interactions with people living with dementia as well as negating stressful situations in applied care settings.

The finding that the CAIT training programme may enhance healthcare professionals' perceived capacity to respond appropriately to emotional changes and identify unmet needs of care recipients could inform strategies on how to ameliorate the onset of distress in people living with dementia using non‐invasive approaches. It has been argued that overt distress can be a way for people living with dementia to express their unmet needs when experiencing difficulties in verbalising person‐centred needs (Cohen‐Mansfield, 2000). The process of providing routine care for people living with dementia who exhibit overt agitation can be highly demanding (Kales et al., 2015) and potentially cause healthcare professionals to experience distress themselves (Hessler et al., 2018). Thus, there is a need to ensure that healthcare professionals have the necessary skills to effectively identify and meet the healthcare needs of people living with dementia in order to negate the onset of distress and ensure the occupational well‐being of carers in dementia care settings (Trivedi et al., 2019). It has been observed that boredom, undesired loneliness, and lack of social interactions may cause underlying unmet needs of people living with dementia who reside in care home settings, which can manifest as overt distress and agitation (Cohen‐Mansfield et al., 2015). The CAIT programme illustrates how the ability to therapeutically communicate can serve as an effective way to learn about the life histories of people living with dementia and inform the implementation of person‐centred care plans that aim to fulfil unmet needs (Estrada, 2021). Furthermore, it has been argued that the process of providing healthcare professionals with opportunities to engage in training programmes that facilitates the application of therapeutic communication techniques may help in the delivery of person‐centred care approaches in dementia care settings (O'Brien et al., 2018). Thus, healthcare professionals' ability to apply communication techniques, as taught on training programmes such as the CAIT course, may help staff in supporting people living with dementia to maintain their psychological needs of comfort, attachment, identity, occupation and inclusion as illustrated in Kitwood's (1997) model of personhood.

While the present study illustrates some of the potential benefits for engaging with the CAIT programme there is a need to acknowledge some limitations when interpreting the results. Firstly, the sample of participants were heterogeneous in terms of job title and position held within dementia care settings. It could be argued that some healthcare professionals have greater contact time with people who have dementia as part of their day‐to‐day professional routine. Thus, some healthcare professions within dementia care may have more opportunities to develop their communication and interaction skills within applied dementia care settings. It would be of interest to assess if there are any differences between healthcare professions, such as Healthcare Assistants, Nurses, and Psychologists, in the communication and interaction skills acquired through engaging in the CAIT programme. This would be useful in ascertaining if there are particular professions within dementia care that would have greater benefit from engaging in the CAIT programme in terms of enhancing therapeutic communication skills and sensitivity towards the healthcare needs of people living with dementia.

Furthermore, the current study did not include the collection of follow‐up data to assess any longer‐term outcomes regarding the effectiveness of the CAIT programme in enhancing healthcare professionals in their perceived communication skills and sensitivity towards identifying the care needs of people living with dementia. It has been argued that training provision alone may not be enough in facilitating healthcare professionals to effectively apply non‐pharmacological interventions and that follow‐up support may be beneficial for healthcare professionals to incorporate the principles covered in a given training programme into their routine care practice (Handley et al., 2017). Previous research has also suggested that the consolidation of skills and information provided on dementia communication skills programmes can be enhanced by follow‐up supervision (Eggenberger et al., 2013). Therefore, further studies are needed to assess how and the extent to which the content covered on a 2‐day CAIT programme are applied in the ongoing practice of healthcare professionals who engage with the training protocol. Furthermore, there may be a necessity to further develop the CAIT programme that provides follow‐up supervision for attendees to re‐enforce the principles and application of therapeutic communication techniques within applied dementia care settings.

## CONCLUSION

CAIT provides up‐to‐date training for frontline healthcare professionals on the application of non‐pharmacological approaches and therapeutic communication techniques when providing care for people living with dementia. The findings of the present study suggested that the 2‐day CAIT programme was beneficial in improving healthcare professionals' confidence in applying their skills to provide effective care for people living with dementia. Furthermore, the CAIT programme was observed to be effective in improving healthcare professionals' perceived skills in therapeutic communications, identifying unmet needs, and responding appropriately to emotional changes of people living with dementia. Whilst healthcare professionals' knowledge of dementia did not significantly increase as a result of engaging in the CAIT programme; this may be due to participants already holding a high level of dementia related knowledge prior to training. Further research is required to assess how healthcare professionals apply the principles of CAIT into their routine practice when providing care for people living with dementia.

## AUTHOR CONTRIBUTIONS

**Sophie Trees:** Formal analysis; writing – review and editing; writing – original draft. **Ian Andrew James:** Conceptualization; writing – review and editing. **Sally Stapleton:** Writing – review and editing; supervision. **Daniel Rippon:** Conceptualization; methodology; formal analysis; supervision; project administration; writing – review and editing; writing – original draft; data curation.

## CONFLICT OF INTEREST STATEMENT

The authors declare no conflict of interest.

## Data Availability

Research data are not shared.
